# Dynamics of the Disparity Vergence Fusion Sustain Component

**DOI:** 10.16910/jemr.12.4.11

**Published:** 2019-12-04

**Authors:** John L. Semmlow, Chang Yaramothu, Tara L. Alvarez

**Affiliations:** Rutgers University, New Brunswick, NJ, USA; New Jersey Institute of Technology, Newark, NJ, USA; New Jersey Institute of Technology, Newark, NJ, USA

**Keywords:** vergence, slow component, fusion sustaining component, vergence feedback control, vergence oscillations

## Abstract

The stereotypical vergence response to a step stimulus consists of two dynamic components: a high velocity fusion initiating component followed by a slower component that may mediate sustained fusion. The initial component has been well-studied and is thought to be controlled by an open-loop mechanism. Less is known about the slow, or fusion sustaining component except that it must be feedback controlled to achieve the positional precision of sustained fusion. Given the delays in disparity vergence control, a feedback control system is likely to exhibit oscillatory behavior. Vergence responses to 4 deg step changes in target position were recorded in eight subjects. The slow component of each response was isolated manually using interactive graphics and the frequency spectrum determined. The frequency spectra of all isolated slow vergence movements showed a large low frequency peak between 1.0 and 2.0 Hz and one or more higher frequency components. The higher frequency components were found to be harmonics of the low frequency oscillation. A feedback model of the slow component was developed consisting of a time delay, an integral/derivative controller and an oculomotor plant based on Robinson’s model. Model simulations showed that a direction dependent asymmetry in the derivative element was primarily responsible for the higher frequency harmonic components. Simulations also showed that the base frequencies are primarily dependent on the time delay in the feedback control system. The fact that oscillatory behavior was found in all subjects provides strong support that the slow, fusion sustaining component is mediated by a feedback system.

## Introduction

The control processes that mediate eye movements face two major challenges: the need for a rapid response despite substantial processing delays, and the need to attain accurate positioning despite errors inherent in neural and muscular mechanisms. These two challenges are best met with different control strategies. Feedback can produce extremely accurate responses, but if delays are present in the feedback loop, response speed must be reduced to keep the system stable. Conversely, open-loop (i.e., preprogrammed) control can generate rapid responses irrespective of processing delays, but these responses will have limited accuracy. Both version and vergence control systems achieve speed and accuracy by combining the two strategies. In version, the two control strategies manifest as separate movements: preprogrammed saccades and feedback-controlled pursuit movements. In vergence, the two control components are less obvious as they merge into a single coordinated response. Nonetheless, considerable evidence supports a “dual mode” control strategy (Semmlow, Hung, & Ciuffreda, 1986) that consists of: an open-loop, pulse-like component that enhances early movement dynamics; and a sustained component that is driven by visual and internal feedback to slowly bring the response to and accurate the final position. (Note, we favor the term “dual-mode” rather than “pulse-step” as it emphasizes the difference in control strategies: open-loop versus feedback.)

**Figure 1:**
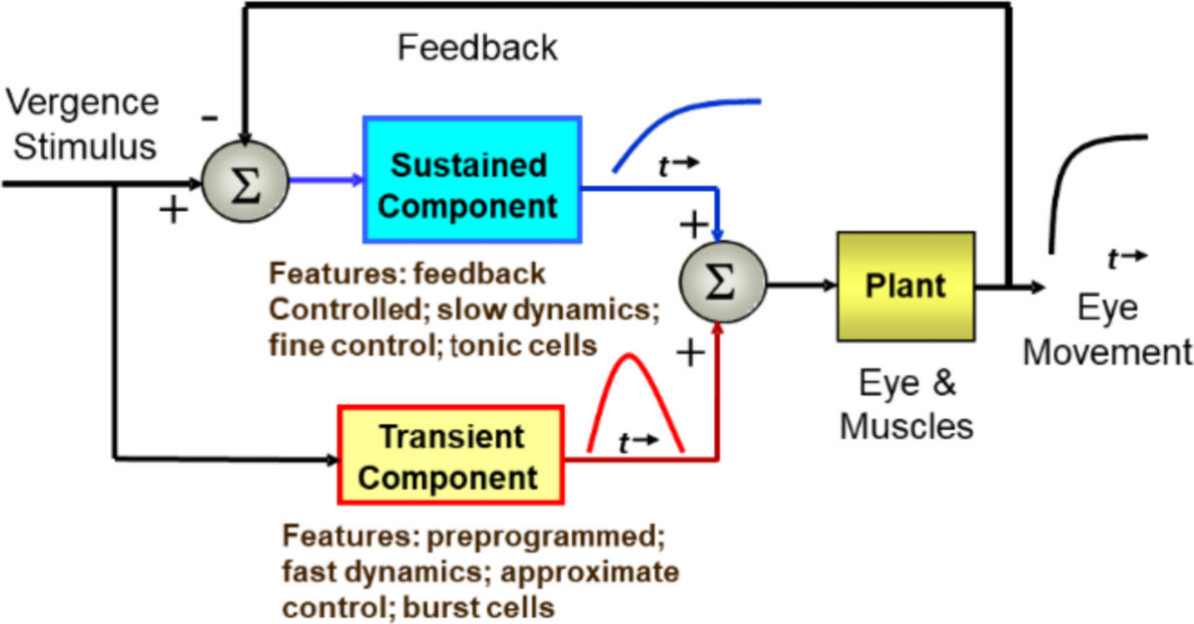
A schematic representation of the dual-mode control strategy showing the control pathways that include an initial, or fusion initiating control component and a slow, or fusion sustaining component.

The neural structures behind these eye movement control components were originally inferred from behavioral data (Alvarez & Kim, 2013; Alvarez, Semmlow, & Yuan, 1998; Alvarez, Semmlow, Yuan, & Munoz, 1999; Alvarez et al., 2010; Horng, Semmlow, Hung, & Ciuffreda, 1998; Jones, 1980; Lee, Chen, & Alvarez, 2008; Semmlow et al., 1986), but have also been identified in neurophysiological studies (Mays, 1984; Mays, Porter, Gamlin, & Tello, 1986). For example, patients with cerebellar stroke, especially those with lesions localized to the cerebellar vermis, can respond to symmetrical vergence step stimuli, but cannot fuse slowly moving vergence ramp or sinusoidal stimuli (Sander et al., 2009). Conversely, patients with lesions to the pontine region show preservation of responses to symmetrical vergence ramps and sinusoids, but impaired initiation of symmetrical vergence step responses (Rambold, Sander, Neumann, & Helmchen, 2005). These clinical findings support dual control of vergence movements consisting of a preprogrammed step and feedback controlled smooth tracking movement. A schematic representation of vergence control is summarized in Figure 1.

A number of sophisticated models have been developed that expand on the simple structure illustrated in Figure 1 (Erkelens, 2011; Maxwell, Tong, & Schor, 2010; Zee, Fitzgibbon, & Optican, 1992; Zee & Levi, 1989). Most of these models emphasize the initial, or fusion initiating component modeled representing it as an open-loop pathway that develops a pulse-like signal. This signal is also referred to as the phasic, pulse, or velocity signal. A model by Erkelens (2011) features a pulse signal, but this component can be altered by feedback and therefore is not truly open loop. Most of these models include additional internal feedback signals usually driven by efference copy (Alvarez, Semmlow, Yuan, & Munoz, 2000). This additional control signal may be essential to achieve the speed and stability of the vergence response (Erkelens, 2011).

The Erkelens’ model has demonstrated appropriate simulations to both step and sinusoidal stimuli, while the model of Maxwell, Tong, and Schor (2010), has accurately simulated behavioral characteristics of both the static and dynamic disparity vergence as well as accommodative vergence (Maxwell et al., 2010). Experimental evidence has shown that the dynamics of initial vergence have a tight coupling between response amplitude and velocity, evidence for a preprogramed control signal that is not influenced by feedback (J. Semmlow, Alvarez, & Granger-Donetti, 2013).

The slow, or fusion sustaining, component has not been as well-studied as the fusion initiating, component (we prefer the term “slow component” since it has not known if this component actually sustains fusion. It is possible that, yet a third component is responsible for fusion lock.) The assumption that this component is under external (i.e., visual) feedback control is strongly supported by empirical data: if this component is responsible for sustained vergence, then the high positional accuracy achieved during binocular fixation (a few minutes of arc) would require feedback. It is impossible to achieve such accuracy from a noisy and variable neurological control system without the use of visual feedback. There may also be an internal feedback pathway that bypasses some of the visual delays to improve stability.

**Figure 2:**
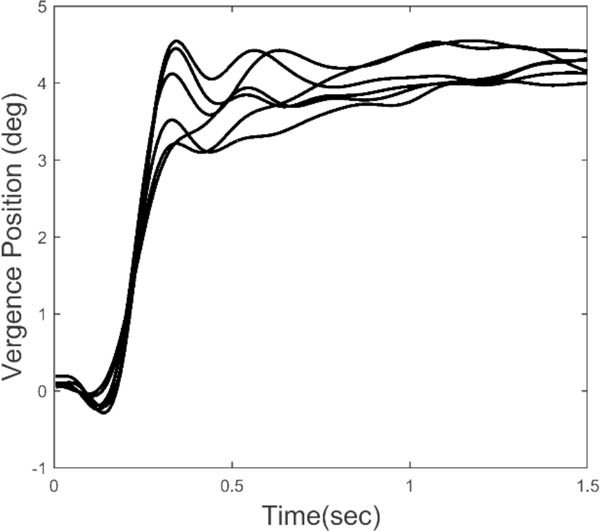
An ensemble of 6 vergence responses to a 4 deg convergent step. The later portion of the responses show what appear to be sustained oscillations.

We know that feedback control systems will exhibit instabilities in the form of oscillatory behavior if the loop gain or loop delay exceed certain limits. Such limits depend on the dynamic characteristics of the process that is being controlled, in this case the oculomotor plant. The essential dynamics of the oculomotor plant have been experimentally determined and can be represented by a second-order system with a relatively long major time constant in the range of 0.2 to 0.4 sec (Robinson, Gordon, & Gordon, 1986). Given that the time delay of a typical vergence response ranges between 0.16 to 0.2 sec, and that the loop gain must be high to achieve small fixation errors, we would expect oscillation to occur during the slow component portion of the response. Figure 2 shows an ensemble of 6 vergence response to a 4.0 deg step change in target distance. After the initial rapid convergence, small, slow oscillatory movements can be seen in the later response, particularly in the time period just following the initial response. These small movements could be due to artifacts in the recording process, but all of the responses we have studied contain sustained oscillatory behavior within a well-defined frequency range. Here, we analyze the dynamic properties of isolated slow component movements to detect and examine oscillatory behavior. We then use standard spectral methods to identify the frequency range of these oscillations. Finally, we apply a simple feedback model to demonstrate the relationship between vergence oscillations and elements of the slow component feedback system.

## Methods

### Subjects:

Eight subjects (3 males and 5 females) between 46 and 72 years of age (57 ± 11.2 years) participated in this study. The average near point of convergence was 9.8 ± 1.9 cm measured from the bridge of the nose while viewing a high acuity target as described in our previous publications (Alvarez et al., 2010; Alvarez et al., 2019; M. Scheiman, Talasan, Mitchell, & Alvarez, 2016; Mitchell Scheiman, Talasan, & Alvarez, 2019). The near (40cm) dissociated phoria measured using a flashed Maddox stimulus was 5 ± 2.5 exophoria (range of 1 eso to 8 exo) and this measurement was also confirmed using our eye movement monitor (Han, Guo, Granger-Donetti, Vicci, & Alvarez, 2010; Kim & Alvarez, 2012; Kim, Vicci, Granger-Donetti, & Alvarez, 2011; Kim, Vicci, Han, & Alvarez, 2011; Santos, Yaramothu, & Alvarez, 2018). All subjects had a normal, uncorrected binocular vision with a stereopsis of < 70 seconds of arc assessed using the Randot Stereopsis Test. All subjects signed written informed consent approved by the New Jersey Institute of Technology Institution Review Board in accordance with the Declaration of Helsinki.

### Recording:

Left and right-eye movements were recorded using an infrared video-based ISCAN (Burlington, MA, USA) eye tracker with a reported accuracy of 0.3° over a ±20° horizontal and vertical range. Symmetrical convergence step stimuli of from 2.0 to 6.0 deg angular vergence demand (i.e., a 4.0 step change in amplitude) were produced using vertical lines projected on two computer screens placed 40 cm from the subject arranged as a haploscope. These lines were driven to produce a step change in vergence demand by a custom software package (Guo, Kim, & Alvarez, 2011). The stimulus was calibrated using real-world targets at known distances and the eye movement monitor was calibrated throughout the experiment using controlled stimuli. Stimulus and data recording were under computer control and eye movements were sampled at 500 Hz using a 12-bit ADC. Approximately 10 to 20 artifactfree recordings were obtained from each subject. Calibration data were taken before and after each movement.

### Analysis:

Vergence responses were computed as the difference between separately calibrated left and right eye movements using the calibration data taken before and after each response. A typical ensemble of vergence movements is shown in Figure 2. Only 4.0 deg step responses were used in this analysis. Velocity was determined using the classic two-point central-difference algorithm. The velocity curve will exhibit oscillations at the same frequencies as the eye movement trace, but enhanced in amplitude.

Isolating the slow component from the total vergence response is the first step in our analysis. While methods have been developed using independent component analysis to separate the initial and slow components, they operate on a group, or ensemble of movements, and identify component averages across the group (Alvarez et al., 2000; Semmlow, Chen, Alvarez, & Pedrono, 2007; Semmlow, Chen, Granger-Donetti, & Alvarez, 2008; Semmlow, Yuan, & Alvarez, 2002). Here, we need to identify the segment dominated by the late response in individual eye movements. Fortunately, the identification of this segment need only be approximate; slight variations will have little effect on the subsequent analysis.

Independent component analysis has shown that the early initiating component is much faster than the sustained component (Alvarez, Semmlow, Ciuffreda, Gayed, & Granger-Donetti, 2007; Castillo et al., 2006; Semmlow et al., 2007, 2002). As the fast, fusion initiating component decays, the sustained or slow component becomes significant and it will alter the velocity profile of the overall response. Therefore, to isolate the slow component, we examine the velocity trace and search for indictors marking a major change in response dynamics.

**Figure 3:**
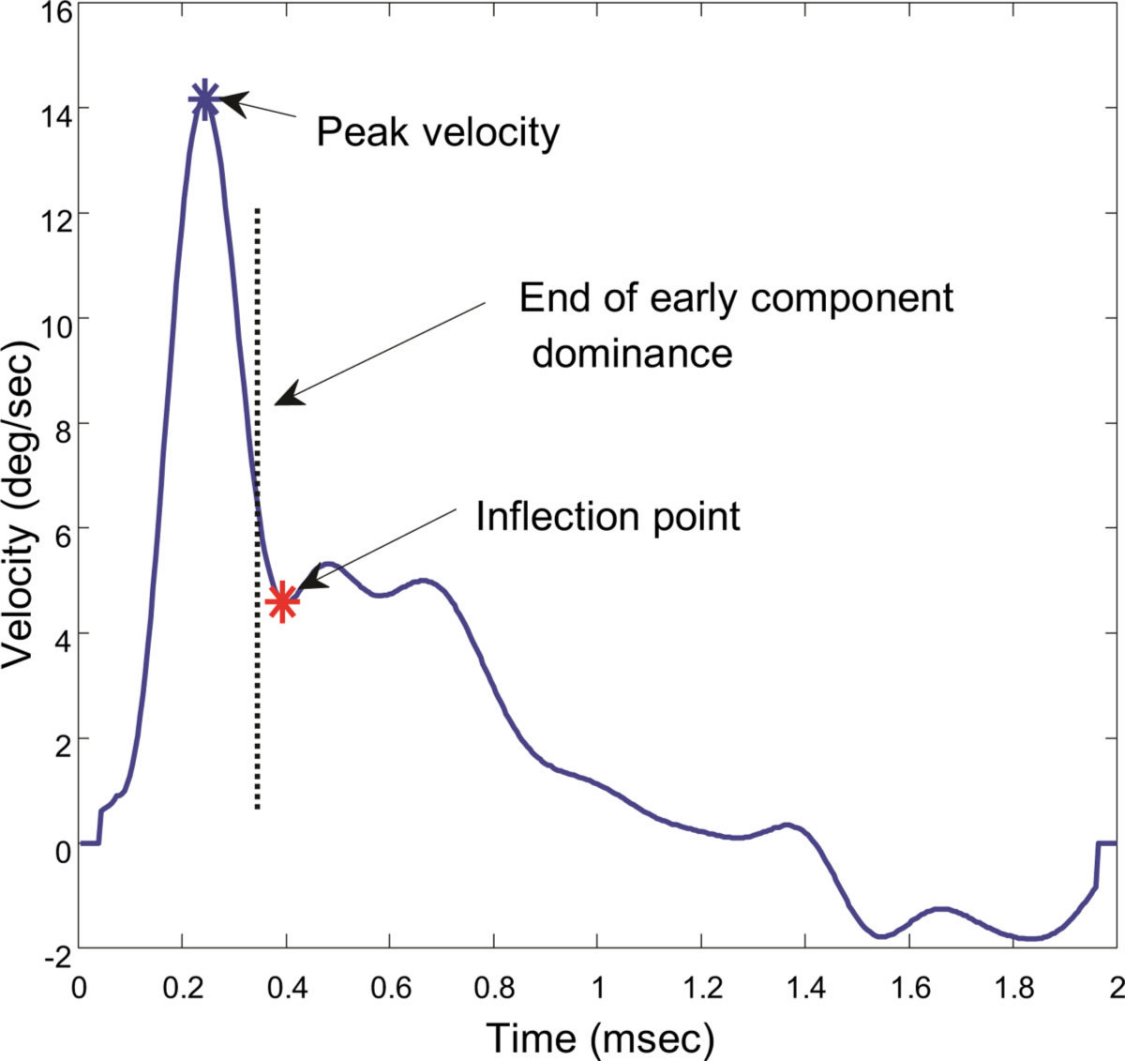
Velocity traces showing the point taken as an inflection and the assumed boundary between the initial and slow component dominant segments. The slow component is taken from this point until the end of the 2.0 sec response. All responses in all subjects showed an inflection point in the velocity trace.

To estimate when the slow component becomes dominant, we search the velocity trace for a point where the smooth downward curve of the velocity trace either reverses or changes slope, Figure 3. Since this is likely the point where the slow component becomes dynamically significant, we isolate the segment beginning at this point and extending until the end of the record. Again, this point is not critical so long as the isolated segment contains a substantial portion of the sustained component and little of the initial, fusion initiating component.

As the isolated segments may contain eye movement positional drift that will produce artifacts in our frequency analysis, we detrend the isolated segments using a quadratic function. A least-squares analysis is used to determine the quadratic function that best fits the isolated segment. This function is then subtracted from the segment. This will reduce the influence of drifts but will not affect oscillatory behavior.

**Figure 4:**
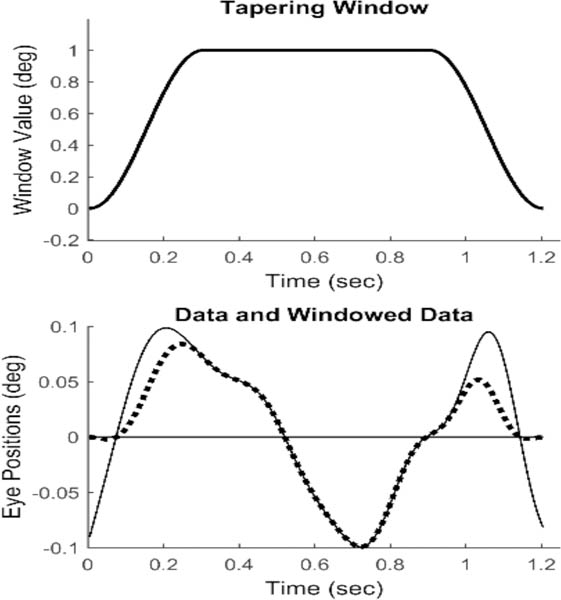
Upper Plot: The Tukey tapering window. Lower Plot: The nonzero end-points seen in the original isolated data (solid line) are reduced to zero (dotted line) after multiplication with the Tukey window.

To obtain slow component frequency characteristics, the Fourier transform was applied to the isolated, detrended segments. The discrete Fourier transform implicitly assumes that the data consist of one cycle of a periodic signal. Discontinuities at the two end-points will produce artifacts in the resulting spectrum. Accordingly, it is common to apply a tapering window to truncated data before applying the Fourier transform. We used the Tukey window shown in Figure 4 (upper plot) to force the segment endpoints to zero, Figure 4 (lower plot, dotted line). This window induces less alteration of the center section of the response.

### Model:

**Figure 5:**
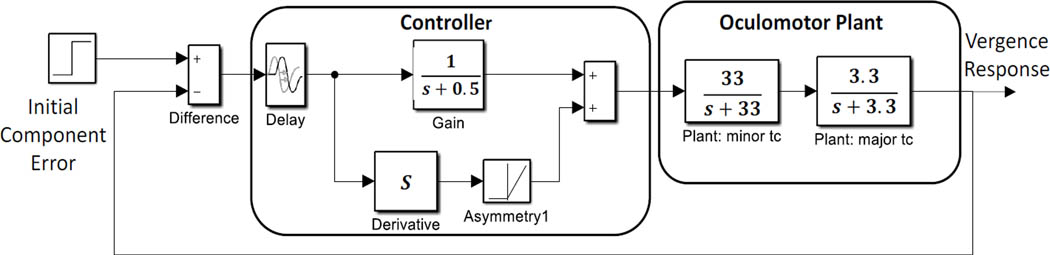
A model of the slow component feedback control system that was used to aid interpretation of the component’s frequency characteristics.

Simulations of a basic feedback control system were used to aid in the interpretation of the slow component frequency characteristics. The model shown in Figure 5 consists of two main sections: the oculomotor plant and neural control processes. The oculomotor plant is based on that developed by Robinson et al. and features two first-order processes having fast and slow time constants (Robinson et al., 1986). The dynamics of the oculomotor plant are determined primarily by the major (i.e., slower) time constant. It was not found necessary to vary this constant during simulations, so it was set as shown in Figure 5 to 0.3 sec (i.e., 1/3.3). This is within the middle of the accepted physiological range of 0.2 to 0.4 sec.

## Results

### Experimental:

**Figure 6:**
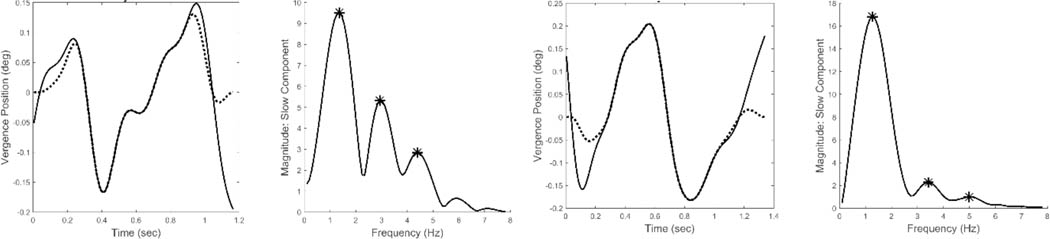
Two examples of isolated slow components (left-hand plots) and their associated frequency spectra (right-hand plots.) Both feature a primary, or fundamental, peak and two or more smaller peaks at higher frequencies. (Peaks are indicated by the * symbol.) This was characteristic of all responses for all subjects. Spectral differences occurred in the frequency of the peaks and the relative magnitudes of the fundamental and secondary peaks.

Figure 6 presents two examples of isolated slow component segments (left-hand plots) and their associated frequency spectra (right-hand plots). The frequency plots show a large peak at around 1.5 Hz and secondary peaks at higher frequencies. representing the response latency. The derivative element contains a direction dependent asymmetry that is responsible for most of the higher harmonics found in the data. With this asymmetry, the derivative element acts like a smaller version of the initial component during the slow component response. The derivative component and its asymmetry may be implemented through internal feedback. The feedforward gain, derivative gain, and response latency could be varied during simulations.

The controller was taken from an early model of the vergence system by Krishnan and Stark (1977) and contains both a derivative element and a feedforward gain in addition to a time delay representing the response latency. The derivative element contains a direction dependent asymmetry that is responsible for most of the higher harmonics found in the data. This combination essentially produces a pulse signal that is proportional to the delayed vergence error. The derivative component and its asymmetry could be implemented using internal feedback. The feedforward gain, derivative gain, and response latency could be varied during simulations.

**Figure 7:**
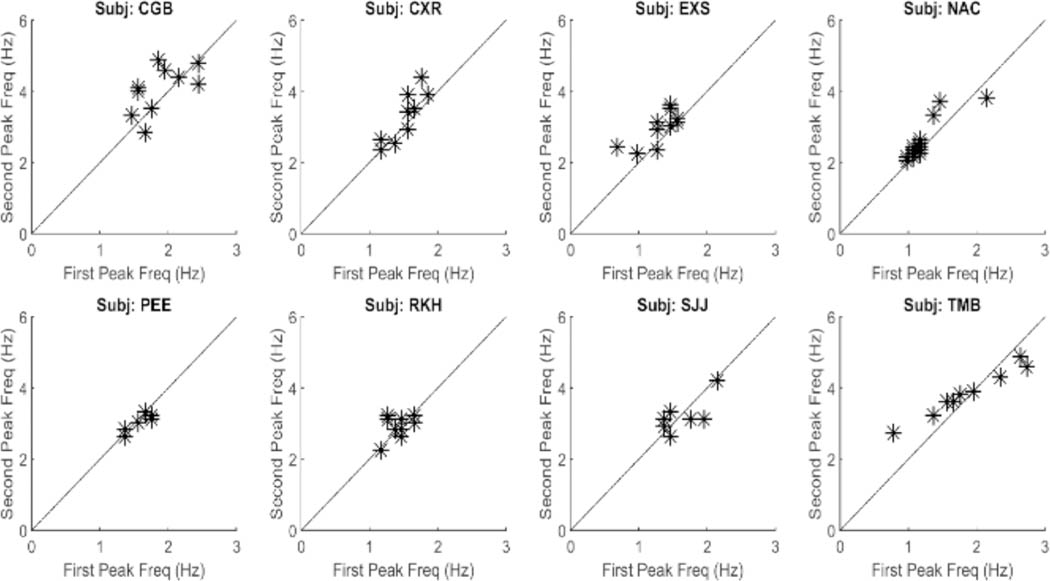
A plot of the frequency of the second spectral peak versus that of the first spectral peak for all responses and all subjects. The points fall close to a straight line with a slope of 2.0 showing that the frequency of the second spectral peak is approximately double that of the first. All correlations between the two parameters were significant at p < 0.01. This suggests that oscillations above those of the fundamental frequency are harmonics probably produced by nonlinearities in the slow component feedback system.

The large primary peaks found in all responses ranged between 1.0 and 2.0 Hz and they indicate the presence of an oscillatory process. These oscillations are likely due to the marginal stability of the slow component feedback system. Examination of the secondary peak frequencies show that they are closely related to the primary peak frequency. Figure 7 is a plot of the frequencies of the second peak versus that of the primary, or fundamental, peak for all spectra found in this study. As seen in Figure 7, the second spectral peak has a frequency that is approximately twice the first. This indicates that the secondary is a harmonic of the fundamental (i.e., first) frequency peak. This was also found to be true of the higher frequency peaks. Such harmonic frequency components are to be expected in a system with nonlinearities.

**Figure 8:**
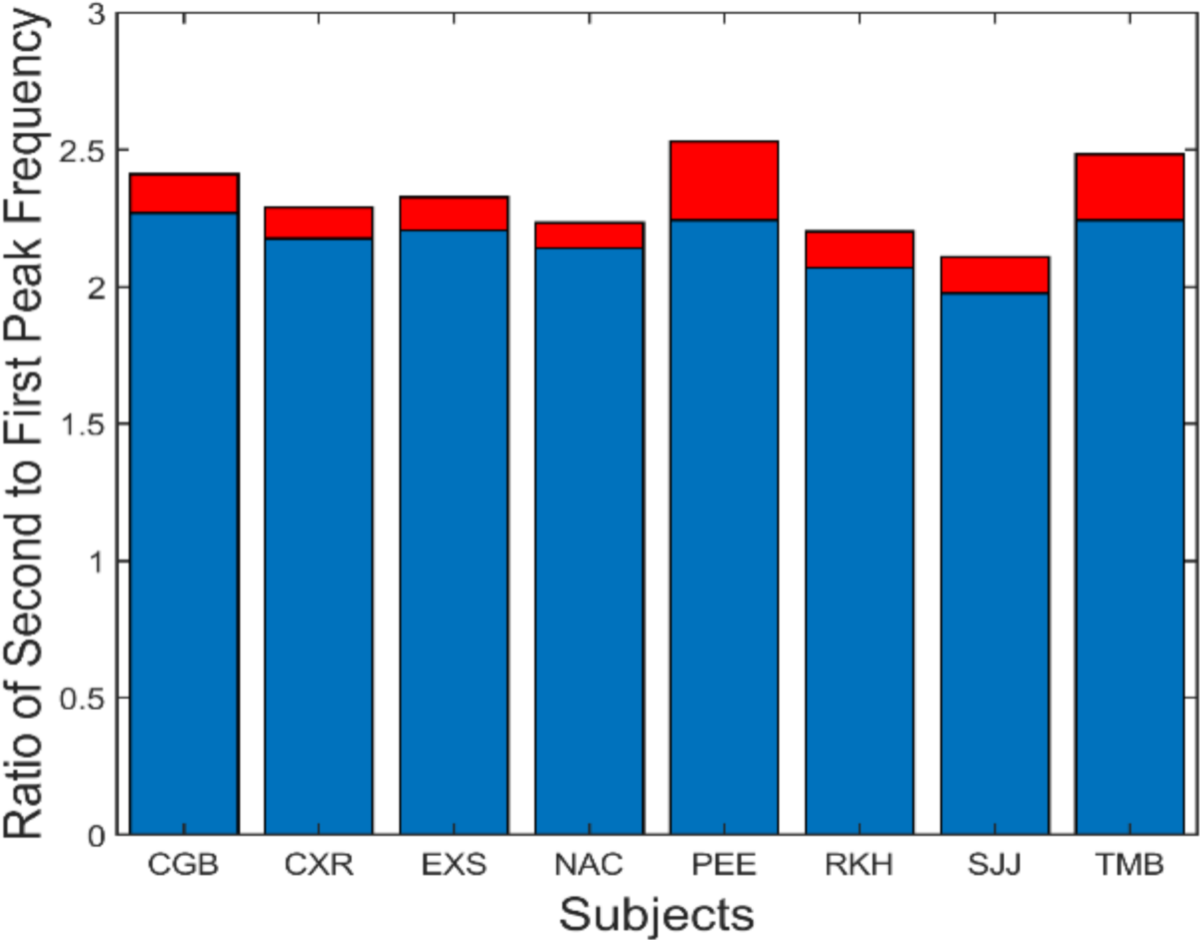
Slow component oscillations showed harmonic components with frequencies slightly more than twice that of the fundamental frequency. Mean is plotted in blue and standard deviations is plotted in red.

A summary of the data in Figure 7 is presented in Figure 8. This figure shows the average (blue) and standard deviations (red) of the ratio of the second peak frequency to that of the fundamental frequency. This protocol is followed in subsequent bar graphs as well. The average of this ratio ranges between approximately 2.2 and 2.4. If the second spectral peak is a harmonic of the fundamental peak, we would expect the ratio to be close to 2.0. However, simulations showed that the nonlinearity in the feedforward pathway produces harmonics that are slightly more than twice the fundamental frequency.

**Figure 9:**
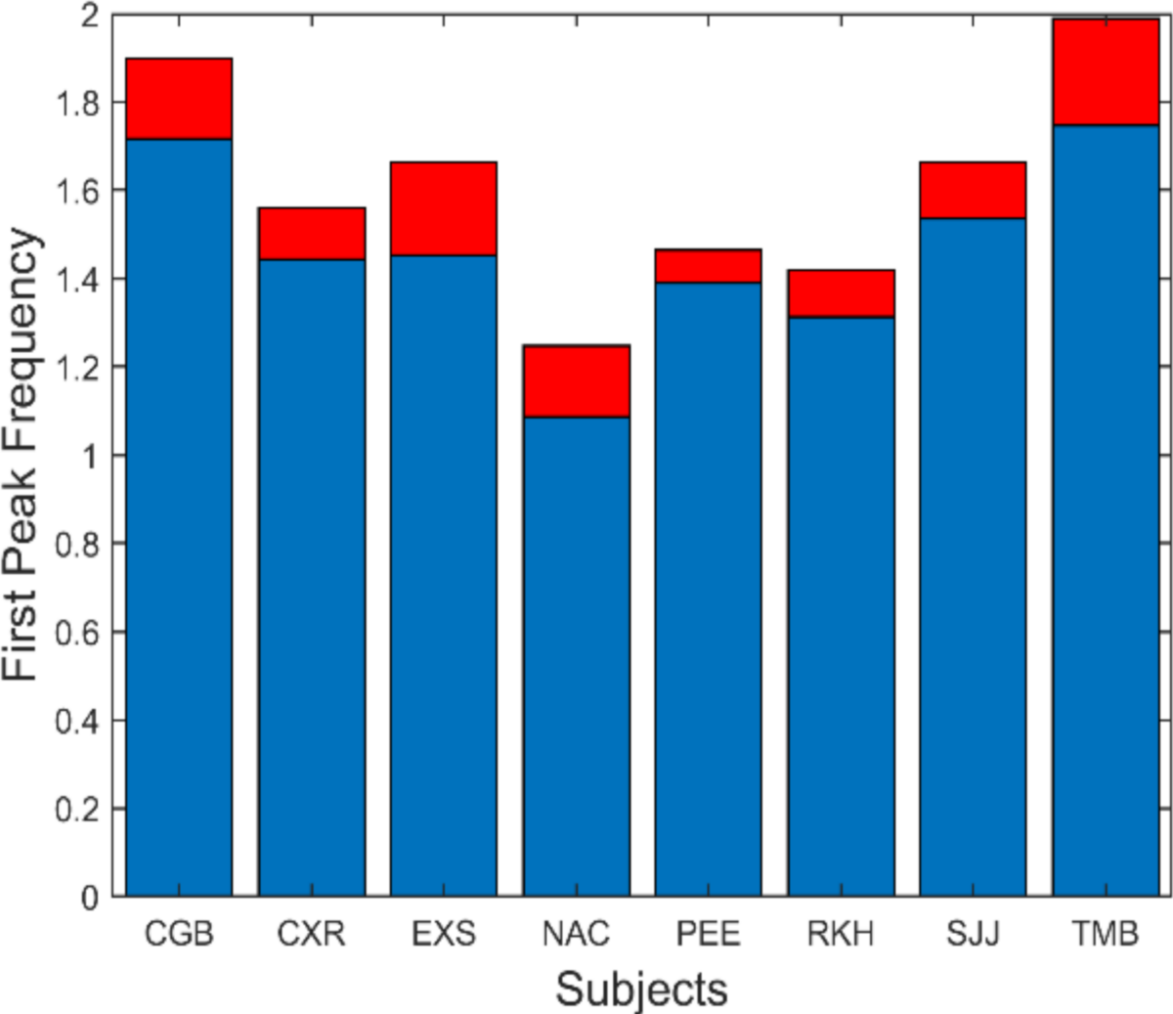
Slow component oscillations had a fundamental frequency that varied between approximately 1.1 and 1.8 Hz. Mean is plotted in blue and standard deviations is plotted in red.

There are several additional measurements that can be extracted from the spectral data. The fundamental peaks indicate the frequency of the primary oscillatory behavior and the value of this frequency is summarized in Figure 9. This figure shows that the average fundamental frequency varies between approximately 1.1 and 1.8 Hz with standard deviations between approximately 0.25 to 0.5 Hz. Considering the various neurological and motor process which must influence the frequency of oscillation, these frequencies are fairly consistent across responses and subjects.

**Figure 10:**
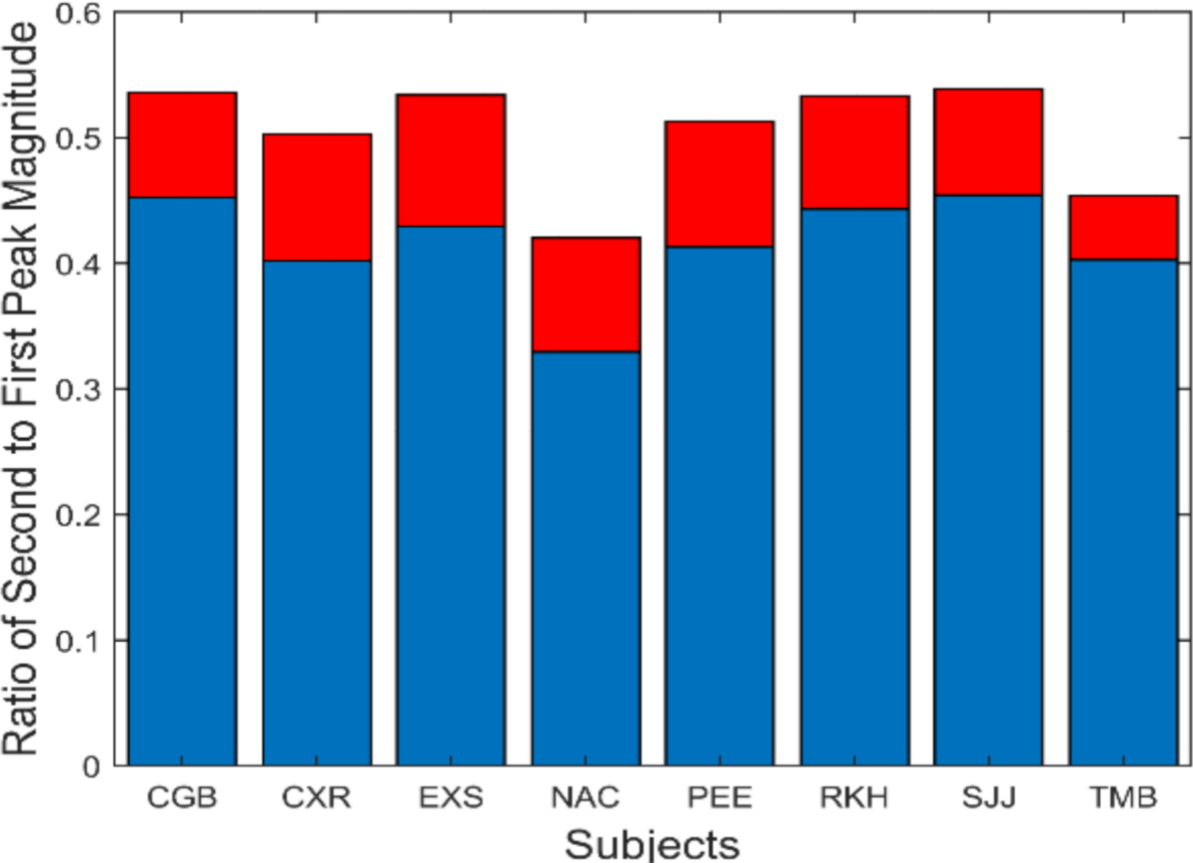
The magnitude of the first harmonic (i.e., second peak) was on average less than half that of the fundamental. Mean is plotted in blue and standard deviations is plotted in red.

Isolated slow component frequency characteristics always included harmonics of the fundamental frequency. The peaks representing these harmonics were smaller than the fundamental peak. The ratio of the magnitude of the second harmonic with respect to the fundamental varied as shown in Figure 10. On average, the second harmonic had a magnitude that was approximately one-half to one-third the magnitude of the fundamental.

**Figure 11:**
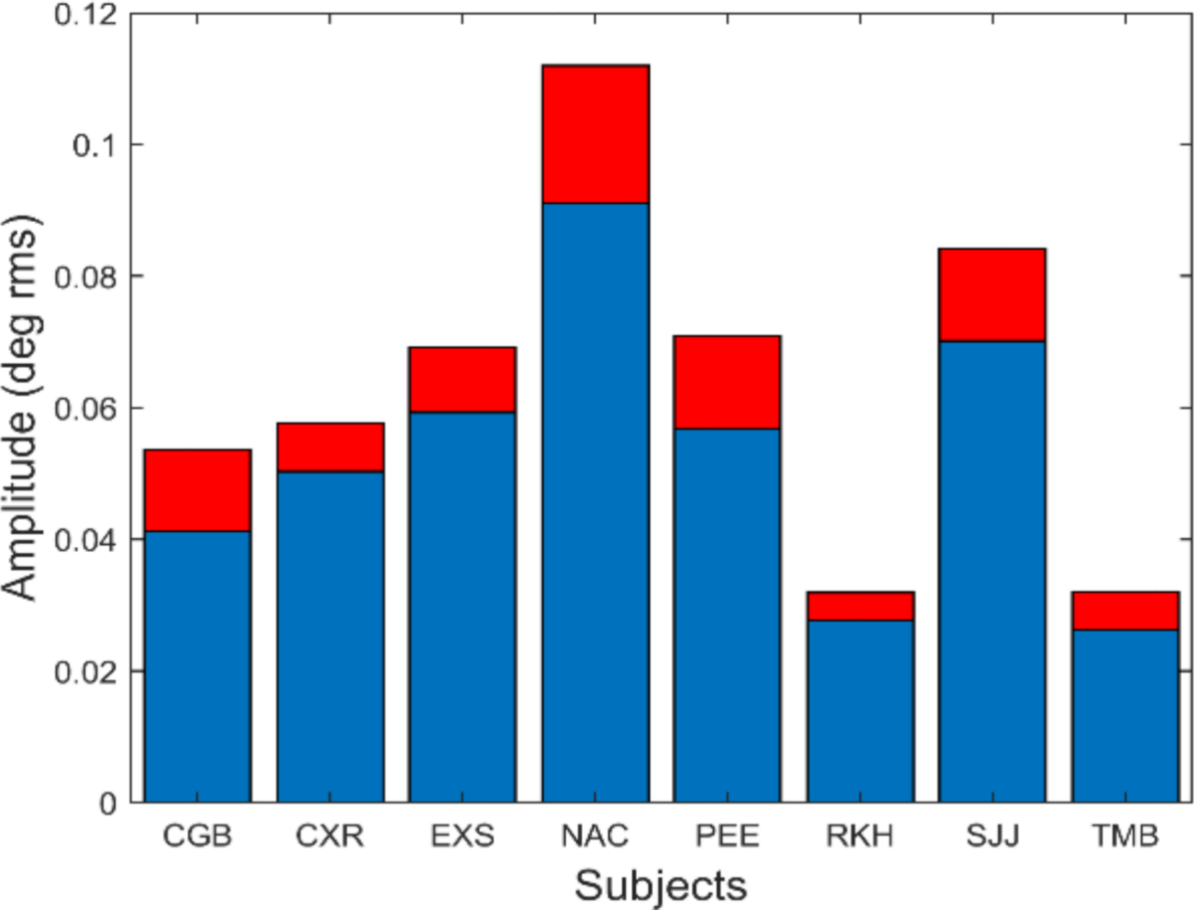
The amplitude of slow component oscillations was small varying between approximately 0.04 and 0.1 deg rms. Peak-to-peak amplitude (not shown) varied between 0.15 to 0.5 deg.

The amplitude of these oscillations is small. Figure 11 shows the average and standard deviation of the root-mean-squared (rms) amplitude of the slow component segment.

In Figure 11, the rms amplitude is seen to vary between 0.025 and 0.09 deg rms. This corresponds to a peak-to-peak variation of between approximately 0.15 and 0.5 deg. Although some of these oscillations were less than the reported (absolute) accuracy of our eye movement monitor, we are only interested in the relative change in position produced by these oscillations which is readily detectable.

To show that these spectral features can be produced by a feedback control system compatible with vergence components, we compare simulations of the model with our experimental results. The range of averages for the four variables shown in Figures 8 to 11 is summarized in Table I and will be helpful in comparing these measurements with model simulations.

### Model Simulations:

The slow component feedback model input takes as its input the vergence error at the end of the initial component, the “Initial Component Error” in Figure 5. Data taken in an earlier study indicated that initial component error varied by roughly ± 0.2 deg for a 4 deg step stimulus, so the input to the model was set to 0.2 deg (J. Semmlow et al., 2013).

The responses obtained through model simulations were analyzed in a manner identical to that used on experimental data. The simulation routine used the same sampling frequency (*f*_*s*_ = 400 Hz) and simulation time responses were windowed using the Tukey window before applying the Fourier transform. The detrend operator was also applied to simulation responses, but since these responses contained no noise or drift, this operation had no effect.

All experimental spectra showed harmonic peaks which are likely caused by nonlinearities in the slow component feedback control system. Nonlinearities that might be expected within the vergence control system include saturations, dead space operators, and different gains and/or dynamics for the convergence and divergence pathways. The latter have been observed experimentally in initial component responses as convergence movements often have different dynamics than divergence movements (Alvarez, Semmlow, & Pedrono, 2005). Nonetheless, preliminary simulations showed that while direction dependent gains or direction dependent time constants could produce higher harmonics, they were never as large as that seen in Figure 6 (upper plot). Similarly, neither saturation elements nor dead space operators, two likely neurological operators, produced significant harmonics. Substantially higher harmonics were created by the derivative asymmetry operator and the gain of this pathway strongly influenced the magnitude of the second harmonic.

**Figure 12:**
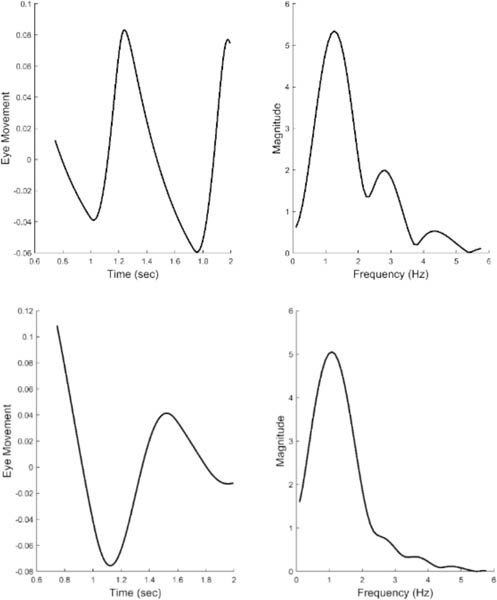
Time responses of model simulations of the slow component response (left-hand plots) and their associated spectral curves (right-hand plots). Only two model parameters were adjusted to produce these two different spectra, the feedforward gain and the derivative gain. The spectra have the same general shape as the those obtained from experimental data and shown in Figure 6. Recall, the two spectra shown in Figure 6 represent the range of shapes found in the experimental data.

Figure 12 shows two different the time responses and spectral curves produced by the model. The two spectra show approximately the same fundamental frequency and higher harmonics as seen in the spectral plots obtained from experimental data and shown in Figure 6. Only the feedforward and derivative gains were modified to produce the two spectra. No attempt was made to match the experimental time responses which would require additional elements to adjust phase shifts, but would not provide any additional information. In addition to varying the feedforward and derivative gains, the value of the time delay could be shifted to match variations in the fundamental frequency. Time delay values required to match the range of fundamental frequencies (1.0 to 2.0 Hz) varied between 0.15 and 0.17 sec. In summary, variation of only three model parameters resulted in spectra that matched the range of spectral shapes found experimentally.

## Discussion

The fact that oscillatory behavior was found in all responses is strong evidence for feedback control in the slow, or fusion sustaining, component of the vergence eye movement response. The fundamental frequency of these oscillations ranged between 1.0 to 2.0 Hz Simulations showed that the time delay element, related to response latency, was most influential in determining the fundamental frequency of the oscillations. Response latencies are known to exhibit both inter-subject and intra-subject variation so latency variation could account for much of the differences observed in the fundamental frequencies. Determining a possible correlation between the fundamental frequency and responses latency will be a part of future studies.

All the model parameters were physiologically reasonable although the time delay element was somewhat less than normal response latencies. However, the initiation of a vergence response would involve additional neurological elements so it is expected that response latency would be longer than the neurological delays in the slow component feedback loop.

The finding that the magnitude of higher harmonics could not be matched by typical nonlinear elements such a direction dependencies and gain nonlinearities (such as saturations and dead-space operators) was surprising. While direction dependent nonlinearities did produce harmonics, the magnitude of these harmonics was less than that seen in some subjects. An asymmetrical derivative element was the only element that was found to produce a substantial second harmonic, although it is possible there are other, untried nonlinearities that are as effective.

An asymmetrical derivative element may seem like an unusual element in the vergence control pathway, but in fact it has the same action as the pulse signal assumed to drive the fusion initiating response. In the slow component neurological control system, small vergence errors would be translated into small unidirectional pulses by the derivative and the amplitude of such pulses would likely be different for convergent versus divergent pulses. While the model used here featured continuous, smoothly varying signals, it is probable that the slow component, like the initial component, is driven through a pulse-like signal. A more realistic model developed around pulse-like signals is another concept for future work.

An extensive search was made for correlations between parameters. For example, the subject with the largest oscillatory behavior, NAC (Figure 11) had the lowest fundamental frequency (Figure 8) and the lowest second harmonic magnitude (Figure 10). However, no statistically significant correlation between these measurements was found when all subjects were included, which may be due to the small sample size. The search for correlations between these parameters and parameters extracted for the initial, fusion initiating response is another subject of continuing study.

## Summary

Oscillatory behavior was found in the isolated slow component segments of all responses in all subjects. A frequency analysis of this behavior showed fairly consistent spectral characteristics with a large lower frequency peak indicating a fundamental frequency and one or more higher frequency peaks. A significant correlation was found between the frequency of the higher frequency peaks and the fundamental frequency indicating that the higher frequency peaks were harmonics of the fundamental frequency. The various spectra differed only in the frequency of the fundamental component and the relative magnitude, and occasionally the number of harmonics.

A simple feedback control model was able to represent the basic spectral features found experimentally. The fundamental frequency was found to be largely determined by the delay element and the harmonics to be generated by an asymmetrical derivative element in the model’s controller. Many candidate nonlinearities were evaluated for production of harmonic frequency components and while many produced some harmonics, the magnitude of these harmonics did not match that found experimentally. The asymmetrical derivative element functions essentially as pulse generator in response to small vergence errors. Such a component might be found in any neurological control system and may, in fact, be a major component in the vergence slow component control system.

## Figures and Tables

**Table 1:** Model verses Experimental Parameters

Variable Name	Range of Experimental Data	Typical Simulation Value
Oscillation Amplitude Figure 11	0.3 – 0.9 (deg rms)	0.6
Fundamental frequency. Figure 9	1.2 – 1.9 (deg)	1.27
Ratio of second to first peak frequency. Figure 8	2.0 – 2.4	2.23
Ratio of second to first peak amplitude. Figure 10	0.3 – 0.5	0.40
